# Editorial: Gut microbiota and its importance on human health - the need for reliable measurements to assess the microbial gut function and its correlated pathologies

**DOI:** 10.3389/frmbi.2025.1657144

**Published:** 2025-07-29

**Authors:** Lilian Terezinha Costa

**Affiliations:** ^1^ Federal University of Rio de Janeiro, Rio de Janeiro, Brazil; ^2^ Faculdade de Medicina de Petrópolis, UNIFASE, Rio de Janeiro, Brazil

**Keywords:** gut microbiota, dysbiosis, biomarker, IBD, microbiome

Understanding the role of human microbiota has shown us that dysbiosis plays an important role in a series of human ailments including chronic gastrointestinal disease, metabolic syndrome, immunity and neurological diseases. However, up to now there is a lack of gut inflammatory biomarkers, which can be used in disease screening, diagnosis, characterization, and monitoring; as prognostic indicators; and for developing individualized therapeutic interventions.

Walking to this Direction this Research Topic has called for measurement tools and applications of gut inflammatory disease biomarkers with potential application in disease screening, diagnosis, characterization, and monitoring; as prognostic indicators; and for developing individualized therapeutic interventions.

Since the beginning of Human Microbiome Project (Nature 569, 641-648 2019) the gut microbiota has play an important role in understanding human health and disease. New research has shown the correlation between microbial composition and physiological processes and pathological conditions, from metabolic disorders and inflammation to neurodevelopment and cancer. However, despite the remarkable progress in microbiome research, a persistent challenge remains: how to obtain reliable, reproducible, and clinically meaningful measurements of gut microbiota structure and function.

This Research Topic, titled *“Gut Microbiota and Its Importance on Human Health – The Need for Reliable Measurements to Assess the Microbial Gut Function and Its Correlated Pathologies,“* aimed to show the state of art of methodological challenges inherent in microbiome science. These contributions provide not only technical insights and innovations but also raise essential questions regarding standardization, reproducibility, and translational applicability.

The Research Topic brings a study on the stability of oral and fecal microbiomes under room temperature storage conditions, providing promising evidence that supports the feasibility of large-scale microbiome studies in real-world settings, even when ideal storage is not possible. The findings have critical implications for the design and logistics of population-based microbiome research.

In another original contribution, microbiome dynamics in response to delta9-tetrahydrocannabinol (THC) treatment are explored in a murine model of obesity. The study demonstrates that specific bacterial features are predictive of weight changes, highlighting the potential of microbiome-informed modeling in metabolic interventions.

Addressing the technical limitations of sampling, a novel approach is introduced via the SIMBA capsule, a minimally invasive device that enables precise spatiotemporal sampling of the small intestine microbiome. This technological innovation opens new avenues for studying host–microbiome interactions in regions previously difficult to access.

The clinical implications of microbiome alterations are further explored in a pilot study linking gut dysbiosis and the reduced abundance of *Faecalibacterium prausnitzii* to pediatric cancer. Although preliminary, these findings point to potential microbial biomarkers for early detection or disease susceptibility.

A population-based study adds to this Research Topic by revealing that Lactobacillus colonization in infants is not only age- and breastfeeding-dependent, but also modulated by specific HLA haplotypes. These results provide critical insights into host genetic influences on microbial ecology, with direct relevance for probiotic design and personalized nutrition strategies.

To deepen our understanding of what constitutes a “healthy” microbiome, a case report on a fecal microbiota super-donor offers a rare glimpse into the complex microbial architecture associated with successful fecal transplantation, reinforcing the value of high-resolution taxonomic profiling.

Finally, a review on intestinal and fecal pH as a fast and accessible biomarker of gut function. The authors argue that pH, often overlooked, can serve as a surrogate for microbial metabolic activity and offers potential as a diagnostic and monitoring tool in clinical practice.

Together, all contributions highlight the multidimensional nature of gut microbiota research—spanning from sample handling and novel measurement tools to mechanistic insights and translational perspectives. Importantly, they converge on a common theme: the urgent need for reliable, standardized methodologies to ensure that microbiome data are not only scientifically robust but also clinically actionable.

Hopefully this Research Topic motivates further discussion, innovation, and clinical research in microbiome. We thank all the authors and reviewers for their valuable contributions and are confident that these works will serve as a meaningful resource for researchers and clinicians alike.

